# Infection of Human Neutrophils With *Leishmania infantum* or *Leishmania major* Strains Triggers Activation and Differential Cytokines Release

**DOI:** 10.3389/fcimb.2019.00153

**Published:** 2019-05-10

**Authors:** Rafeh Oualha, Mourad Barhoumi, Soumaya Marzouki, Emna Harigua-Souiai, Melika Ben Ahmed, Ikram Guizani

**Affiliations:** ^1^Laboratory of Molecular Epidemiology and Experimental Pathology - LR16IPT04, Institut Pasteur de Tunis, Université de Tunis El Manar, Tunis, Tunisia; ^2^Faculté des Sciences de Bizerte, Université de Carthage, Tunis, Tunisia; ^3^Laboratory of Transmission, Control and Immunobiology of Infections - LR16IPT02, Institut Pasteur de Tunis, Université de Tunis El Manar, Tunis, Tunisia

**Keywords:** *Leishmania* spp., neutrophils, oxidative burst, degranulation, DNA release, apoptosis, multiplex cytokine bead array, amastigote survival

## Abstract

Leishmaniases are neglected diseases, caused by intracellular protozoan parasites of the *Leishmania* (*L*.) genus. Although the principal host cells of the parasites are macrophages, neutrophils are the first cells rapidly recruited to the site of parasites inoculation, where they play an important role in the early recognition and elimination of the parasites. The nature of early interactions between neutrophils and *Leishmania* could influence the outcome of infection. Herein we aimed to evaluate whether different *Leishmania* strains, responsible for distinct clinical manifestations, could influence *ex vivo* functional activity of neutrophils. Human polymorphonuclear leukocytes were isolated from 14 healthy volunteers and the *ex vivo* infection of these cells was done with two *L. infantum* and one *L. major* strains. Infection parameters were determined and neutrophils activation was assessed by oxidative burst, degranulation, DNA release and apoptosis; cytokine production was measured by a multiplex flow cytometry analysis. Intracellular amastigotes were rescued to determine *Leishmania* strains survival. The results showed that *L. infantum* and *L. major* promastigotes similarly infected the neutrophils. Oxidative burst, neutrophil elastase, myeloperoxidase activity and apoptosis were significantly increased in infected neutrophils but with no differences between strains. The *L. infantum*-infected neutrophils induced more DNA release than those infected by *L. major*. Furthermore, *Leishmania* strains induced high amounts of IL-8 and stimulated the production of IL-1β, TNF-α, and TGF-β by human neutrophils. We observed that only one strain promoted IL-6 release by these neutrophils. The production of TNF-α was also differently induced by the parasites strains. All these results demonstrate that *L. infantum* and *L. major* strains were able to induce globally a similar *ex vivo* activation and apoptosis of neutrophils; however, they differentially triggered cytokines release from these cells. In addition, rescue of intracellular parasites indicated different survival rates further emphasizing on the influence of parasite strains within a species on the fate of infection.

## Introduction

Leishmaniases are a complex group of neglected diseases, caused by the intracellular protozoan parasites of the genus *Leishmania*. They are endemic in more than 98 countries and cause significant morbidity and mortality worldwide. They are characterized by a spectrum of clinical manifestations of the disease ranging from the self-healing skin lesions of cutaneous leishmaniasis (CL) to the visceral leishmaniasis (VL) that is fatal in the absence of treatment. The different clinical manifestations depend on the *Leishmania* parasite species and on the immune response of the host among other factors (Herwaldt, 1999; Guizani et al., 2011; Alvar et al., 2012). An estimated 700,000 to 1 million new cases and some 26,000 to 65,000 deaths occur annually (Who, 2019). In addition, the Global Burden of Disease 2017 study estimated the prevalence of leishmaniases to be 4.130 Million (95 % Uncertainty Interval 3.515–4.966) (Diseases, 2018). Maps presenting the global distribution of these diseases and their local risk factors were recently updated; based on such maps computer modeling predicted that 1.7 billion are living in areas at risk for leishmaniases (Pigott et al., 2014). In spite of sustained efforts, no effective human vaccine is yet available (Kumar and Engwerda, 2014; Didwania et al., 2017; Seyed et al., 2018). The mainstay therapy is based on the use of pentavalent antimonials, which present adverse effects and increasingly induce drug resistance (Hefnawy et al., 2017; Ghorbani and Farhoudi, 2018). Studies in animal models have shown that protection against the disease is associated with the production of IL-12 by innate cells, which induces the proliferation of CD4+ Th1 cells, which in turn produce IFN-γ to activate macrophages to kill the parasites (Sacks and Noben-Trauth, 2002; Kaye and Scott, 2011). Furthermore, it seems that early events that occur during the establishment of the infection in the skin are very important to the development of an effective immune response against *Leishmania* infection (Peters and Sacks, 2006). Infectious *Leishmania* promastigotes are inoculated to the mammalian host by sand fly bites, then these parasites transform into amastigotes inside parasitophorous vacuoles within a range of host cells: macrophages, dendritic cells and neutrophils as result of complex host/pathogen/ vector interactions (Rodríguez and Wilson, 2014; Martínez-López et al., 2018). Studies on animal models have shown that neutrophils are massively and rapidly recruited to the site of infection and are the first cells to encounter the parasites (Müller et al., 2001; Peters et al., 2008). Neutrophils constitute the first line of defense against these pathogens. They participate in their elimination by several mechanisms including the production of reactive oxygen species (ROS), the release of azurophilic granules that contain antimicrobial proteins such as Neutrophil Elastase (NE) and myeloperoxidase (MPO) (Segal, 2005; Nauseef, 2007). In addition, neutrophils can release extracellular traps (NETs) composed of histones, fibrous DNA and granule proteins (Brinkmann et al., 2004), which can trap extracellular pathogens and in some cases kill them (Kolaczkowska and Kubes, 2013; Bardoel et al., 2014). The role of neutrophils in host defense against leishmaniases has been well studied in animal models. Both protective and non-protective roles against *Leishmania* infection have been reported for these cells, which depend on *Leishmania* species and host immune responses (Peters and Sacks, 2009; Charmoy et al., 2010; Ribeiro-Gomes and Sacks, 2012; Carlsen et al., 2015b; Hurrell et al., 2016). Indeed, it was shown that at the time of *L. major* infection, depletion of neutrophils in susceptible BALB/c mice reduced the parasite load and induced resistance to *L. major* infection (Tacchini-Cottier et al., 2000). In contrast, at the same time, the depletion of neutrophils in resistant C57Bl/6 exacerbated parasite load and footpad lesion (Tacchini-Cottier et al., 2000; Ribeiro-Gomes et al., 2004; Chen et al., 2005). The use of neutropenic *Genista* mice that lack mature neutrophils has provided further information about the role of neutrophils in disease progression. Indeed, these mice were able to control parasite load and resolve their lesion after *L. mexicana* infection suggesting that neutrophils impaired the development of effective immune response against this species (Hurrell et al., 2015). The neutrophils can also influence the development of the immune response against *Leishmania* by secreting cytokines and chemokines. These cytokines can influence the subsequent T cell differentiation (Tacchini-Cottier et al., 2000). In addition, the chemokines can attract other innate immune cells to the site of infection where they interact with neutrophils, which can influence the early anti-*Leishmania* response (Ribeiro-Gomes and Sacks, 2012; Hurrell et al., 2016). *Ex vivo* studies have shown that the impact of neutrophils on parasite survival depends on the *Leishmania* species. Indeed, *L. major* can escape killing by neutrophils, which act as “Trojan horses” providing to the parasites a silent entry into macrophages (Van Zandbergen et al., 2004; Ritter et al., 2009). Furthermore, the NETs release induced *in vitro* can trap the parasites but could kill them or not (Guimarães-Costa et al., 2009; Gabriel et al., 2010). Thus, we believe that understanding the interaction between *Leishmania* species and neutrophils could help understanding the mechanisms controlling these parasites. In this context, as a first step we aimed to evaluate whether *Leishmania* strains that belong to *L. infantum* and *L. major* species, responsible for distinct clinical manifestations in the Old World, could influence *ex vivo* functional activity of human neutrophils. The interactions were addressed by the characterization of infection parameters (percentage of infection, index of infection), by measuring the oxidative burst, degranulation, neutrophils extracellular traps (NETs) release and apoptosis. The cytokine production was also measured by a multiplex flow cytometry analysis. Viability of intracellular parasites was also assessed by an MTT assay on rescued amastigotes.

## Materials and Methods

### Ethical Statement

Blood sample collection was done from fourteen informed healthy volunteers that consented by writing to participate to the study. The Ethical committee of the Institut Pasteur de Tunis approved this study (2018/07/I/LR11IPT04).

### Parasites

Three laboratory strains were used in this study: *Leishmania (L*.) *infantum* LV50 (MHOM/TN/94/LV50) was isolated from a visceral leishmaniasis case, *L. infantum* Drep-14 (MHOM/TN/96/Drep-14) from the lesion of a sporadic cutaneous leishmaniasis patient (CL patient only), and *L. major* Empa-12 (MHOM/TN/2012/Empa-12) from a zoonotic cutaneous leishmaniasis case. Virulence of these parasites was maintained by regular passages through BALB/c mice. Mice were subcutaneously (s.c.) infected with 1 × 10^6^ *L. major* (Empa-12) stationary phase promastigotes. While 1 × 10^7^ *L. infantum* strains Drep-14 or LV50 stationary phase promastigotes were injected intravenously (i.v.) in the lateral tail vein of BALB/c mice. The *L. major* Empa-12 strain was isolated from the infected footpad lesion. While *L. infantum* LV50 and Drep-14 strains were isolated from the mouse inguinal lymph node. Samples taken from the lesion (in the case of *L. major*) or lymph nodes (in the case of *L. infantum*) were cultured at 22 °C in RPMI-1640/ Glutamax medium (Gibco BRL, Germany) containing penicillin (100 U/mL) and streptomycin (100 μg/mL) supplemented with 10 % heat-inactivated Fetal Bovine Serum (FBS) (Gibco BRL, Germany). Cultures were monitored every 3–4 days for the presence of flagellated promastigotes forms by microscope. The growing parasites were cryopreserved to constitute the stocks used in this study. The growth kinetics of each strain was established to determine the stationary growth phase. Promastigotes at this phase were used in the infection experiments.

### Polymorphonuclear Neutrophil (PMN) Isolation and Purification

Neutrophils granulocytes from fourteen healthy volunteer donors were isolated based on density gradient centrifugation using Ficoll-Paque density gradients and dextran sedimentation as previously described (Kuhns et al., 2015). Briefly, with no delay between sampling and purification, platelet-rich plasma was removed from EDTA-anticoagulated (BD Vacutainer, BD Bioscience, UK) blood by centrifugation at 500 g for 10 min. The blood cells then were overlayed on Ficoll-Paque (GE Healthcare, Sweden) and the mononuclear cells were aspirated and eliminated after centrifugation at 500 g for 30 min at +4 °C. The red blood cells were separated from the neutrophils by sedimentation for 35 min at room temperature in 6 % dextran (Sigma, Denmark). The neutrophils rich supernatant was collected by centrifugation at 300 g at +4 °C. The remaining red blood cells were removed by a hypotonic lysis Buffer (Sigma, Denmark). Finally, the neutrophils were collected and washed two times with phosphate buffer saline (PBS) and centrifuged at 300 g for 5 min. The viability counts of the PMNs were systematically checked by trypan blue dye exclusion [0.4 % trypan blue solution (Sigma)]; viability was estimated as > 99 %. The purity of granulocytes was > 97 % as determined microscopically by morphological analysis after May-Grunwald-Giemsa staining with RAL 555 Kit (RAL DIAGNOSTICS, France). The Supplementary Table 1 summarizes the contribution of the donors to the experiments.

### *Leishmania* Infection of PMN

2 × 10^6^ PMN were cultured in 24 wells plates for 18 h at 37 °C and 5 % CO_2_ in RPMI-1640/ Glutamax medium containing penicillin (100 U/mL) and streptomycin (100 μg/mL) supplemented with 5 % FBS, in the presence or absence of stationary phase *Leishmania* promastigotes at a ratio of 10 parasites per 1 neutrophil (Multiplicity Of Infection (MOI) 10 or otherwise as indicated). After 18 h of incubation, the cultures were washed to remove extracellular parasites. Cells were then fixed and stained with RAL 555 kit (RAL DIAGNOSTICS, France) following the manufacturer's instructions. The numbers of infected cells and intracellular amastigotes within infected cells were quantified by counting at least 100 cells under optical microscopy. Infection index was calculated as: the percentage of infected cells x mean amastigotes number per cell.

### Measurement of Oxidative Burst

Superoxide anion (O_2_^**−**^) production was measured using a colorimetric nitroblue tetrazolium (NBT) assay, in which the soluble yellow dye NBT is reduced by intracellular O_2_^**−**^ generated upon activation of phagocytes forming insoluble blue black formazan crystals. The oxidative burst assay was performed as described previously (Marques et al., 2015). Briefly, the neutrophils and the infected neutrophils, or the positive control neutrophils that were stimulated with 100 nM Phorbol 12-Myristate 13-Acetate (PMA), were incubated in triplicate in 1 mL of RPMI-1640/ Glutamax medium containing penicillin (100 U/mL) and streptomycin (100 μg/mL) supplemented with 5 % FBS containing 0.2 % NBT (Sigma, China), at 37 °C and 5 % CO_2_ for 18 h, or at different time points: 1, 2, 3, 4, 5, 6, and 18 h when we performed a kinetic analysis of O_2_^**−**^ production. Following this incubation, the cells were washed with warm PBS and the NBT deposited inside the PMNs was solubilized with 10 % SDS and 0.1 N HCl. Absorbance of dissolved NBT solution was measured at 570 nm using a microplate reader (MULTISCAN GO, Thermo Scientific, Finland). Results are expressed as the mean values of ${\text{O}}_{2}^{-}$ production from 14 donors ± standard deviation (SD).

### Degranulation Assays

Myeloperoxidase (MPO) and elastase activity of neutrophils were measured spectrophotometrically in supernatants of PMN and PMN infected with *Leishmania* parasites during 18 h, by adding specific substrates. Neutrophils stimulated with 100 nM PMA were used as a positive control. When we did kinetics of enzymes release, we incubated the cells for 2, 4, 6, and 18 h. The activity of MPO was assessed in the supernatants as described previously (Kumar et al., 2002). Briefly, 100 μL of the substrate cocktail containing *o*-Dianisidine/H_2_O_2_ was added to 100 μL of culture supernatants. The mixture was kept at room temperature for 10 min and the absorbance of oxidized *o*-Dianisidine was measured at 450 nm. Elastase activity was quantified by addition of 100 μL of 1mM elastase substrate [N-methoxysuccinyl-Ala-Ala-Pro-Val p-nitroanilide (Sigma, USA)] to 100 μL of supernatants (Marques et al., 2015). After 3 h of incubation at 37 °C, absorbance of the cleaved p-nitroanilide was measured at 405 nm in a microplate reader (MULTISCAN GO, Thermo Scientific, Finland). Results are expressed as mean values of MPO and elastase production from 14 donors ± standard deviation (SD).

### Quantification of DNA Released in the Culture Medium

Neutrophils were incubated with *Leishmania* promastigotes as described above or with 100 nM PMA as positive control, for 18 h. To quantify the release of DNA (hallmark of NET release) into the culture supernatant, *EcoR1* and *HindIII* (20 U/mL) restriction enzymes (GE Health care, Amersham, Greece) were added to the cultures as described (Guimarães-Costa et al., 2009) to digest any released DNA, and were incubated for additional 4 h at 37 °C. Then, the cells were centrifuged and DNA release was quantified in the culture supernatants, using the Qubit 1x double stranded HS Assay kit (Invitrogen, USA), by Qubit 4 Fluorometer following the manufacturer instructions. Results are expressed as mean values of NETs DNA release from seven donors ± standard deviation (SD). To visualize the DNA, we also did electrophoresis on 0.7 % agarose gels in presence of ethidium bromide (0.5 μg/mL) at 40 V/cm, and observation under UV light.

### Measurement of Neutrophils Apoptosis

For the detection of apoptotic or necrotic cell death, the PE-Annexin V / 7-amino-actinomycin D (7-AAD) apoptosis detection kit (BD Biosciences, San Diego, CA) was used according to the manufacturer's protocol. Briefly neutrophils (1 × 10^6^ cells/mL) were incubated in the presence or absence of promastigotes (MOI of 10). After 18 h of incubation, the extracellular parasites were removed as described above. Then, the PMNs were washed with cold PBS and stained with PE-Annexin V / PerCP-cy5.5-(7-AAD). After 15 min of incubation in the dark at room temperature, the cells were re-suspended in 1x binding Buffer and the samples were acquired by flow cytometry (FACS Canto II, BD Biosciences).

### Cytometric Bead Array Assay (CBA)

Interleukin-8 (IL-8), interleukin-1β (IL-1β), interleukin-6 (IL-6), interleukin-10 (IL-10), tumor necrosis factor alpha (TNF-α), and interleukin-12p70 (IL-12p70) protein levels were detected and quantified in culture supernatants of neutrophils infected or not by *Leishmania* promastigotes after 18 h of incubation at 37 °C in the presence of 5 % CO_2_, by multiplex flow cytometry using the BD Cytometric Bead Array (CBA): Human Inflammation Cytokines kit according to the instructions of the manufacturer (BD Biosciences, San Diego, CA). Briefly, 50 μL of mixed antibody conjugated capture beads were incubated with 50 μL supernatants or cytokine standard dilutions containing a mixture of each recombinant protein, and with 50 μL of phycoerythrin (PE)-conjugated detection antibodies. After 3 h of incubation, the mixture was washed, centrifuged and re-suspended in 300 μL of wash buffer. Finally, fluorescence signals of the beads were acquired by flow cytometry (FACS Canto II, BD Biosciences, San Diego, CA). Results were expressed as the cytokine concentration obtained for each of ten tested donors.

### ELISA Quantification of TGF-β

Active form of transforming growth factor beta (TGF-β) levels were quantified, in culture supernatants of non-infected and infected cells collected after 18 h of incubation, by Enzyme-linked immunosorbent assay (ELISA) using Human TGF-β ELISA Sets (BD Biosciences, San Diego, CA) according to manufacturer's instructions. All supernatants were activated by acidification using 1 N HCl and incubated for 60 min at +4 °C. The activated samples were then neutralized with 1 N NaOH as recommended by manufacturer's instructions. The TGF-β concentrations in the supernatants were interpolated from a standard curve using known amounts of the recombinant cytokine. Results were expressed as the mean cytokine concentration of technical replicates, obtained for each of seven tested donors.

### Statistical Assessment and Principal Component Analysis

Most graphs were prepared using GraphPad Prism version 7.0 (GraphPad Software). Data were shown as mean values ± standard deviation (SD). The non-parametric Mann–Whitney test was used to assess the differences between two groups. These differences were considered significant when the *p*-value was < 0.05. ^*^p < 0.05, ^**^p < 0.01, ^***^p < 0.001 on the figures indicate statistically significant differences at the indicated *p*-values.

Principal component analysis (PCA) was performed using R3.4 under the RStudio environment. Since PCA can only be performed on complete data, we considered through this analysis data collected from healthy donors, out of the fourteen, for which data was available for all the observations. Thus, data collected from ten donors about the infection parameters and the cytokine production under the non-infected (NI) and the infected conditions were formatted into a data frame. This led to 40 individuals (10 donors in 4 conditions) for which 11 variables were observed (infection index, percentage of infection, Oxidative burst, Elastase, MPO, IL-12p70, IL-8, IL-6, IL-10, IL1-β, and TNF-α). The observations “TGF-β” and “NETs” were discarded in the analysis as it only concerned seven donors and no missing data could be considered for PCA. An additional observation, called “species,” was used as a supplementary qualitative variable to generate the groups of individuals according to the infection condition (NI, Drep-14, LV50, and Empa-12). The package *FactoMineR* was used for PCA calculation and the package *factoextra* was used to generate the corresponding figures.

### Measure of Neutrophils Leishmanicidal Activity Against Intracellular *L. infantum* and *L. major*

To evaluate the impact of neutrophils activity on intracellular *Leishmania* survival, promastigotes of each strain were incubated with an excess of neutrophils at a ratio of 1 parasite per 10 neutrophils to allow efficient parasite internalization as described previously (Carlsen et al., 2015a). Briefly, 2 × 10^6^ neutrophils isolated from three donors were infected with 0.2 × 10^6^ promastigotes (MOI of 0.1) in technical replicates in 24 well-plates. After 18 h of infection, the cultures were washed to remove the extracellular parasites. Cells were then fixed and stained with RAL 555 kit, and the infected cells and the intracellular amastigotes per infected cells were quantified by counting at least 100 cells under optical microscope. The remaining cells were lysed by SDS (0.01 %), and the pellets were washed with PBS and resuspended in 100 μL Schneider medium supplemented with 10 % FBS and cultured at 22 °C in 96 well-plates. Parasites viability was assessed right after the lysis of the cells and 24 h later, by a Methylthiazolyldiphenyl-tetrazolium bromide (MTT) assay as described previously (Harigua-Souiai et al., 2018). Briefly, 20 μL of MTT (5 mg/mL) were added to each well and incubated at 22 °C. After 4 h of incubation, the MTT is reduced to formazan crystals by the mitochondrial dehydrogenases of the live parasites, and 150 μL of DMSO was added to dissolve the formazan crystals. Absorbance was measured at 570 nm using a microplate reader (MULTISCAN GO, Thermo Scientific, Finland). A range of serial dilutions of counted promastigotes (1:2) were performed and submitted to MTT assays. The resulting linear equation was used to interpolate the number of parasites recovered from the infected PMNs, having an active metabolism and thus which are viable. Results are expressed as mean number of viable parasites ± standard deviation (SD).

## Results

### Human Neutrophils Similarly Uptake *Leishmania* Promastigotes From Different Strains

**Figure 1:**
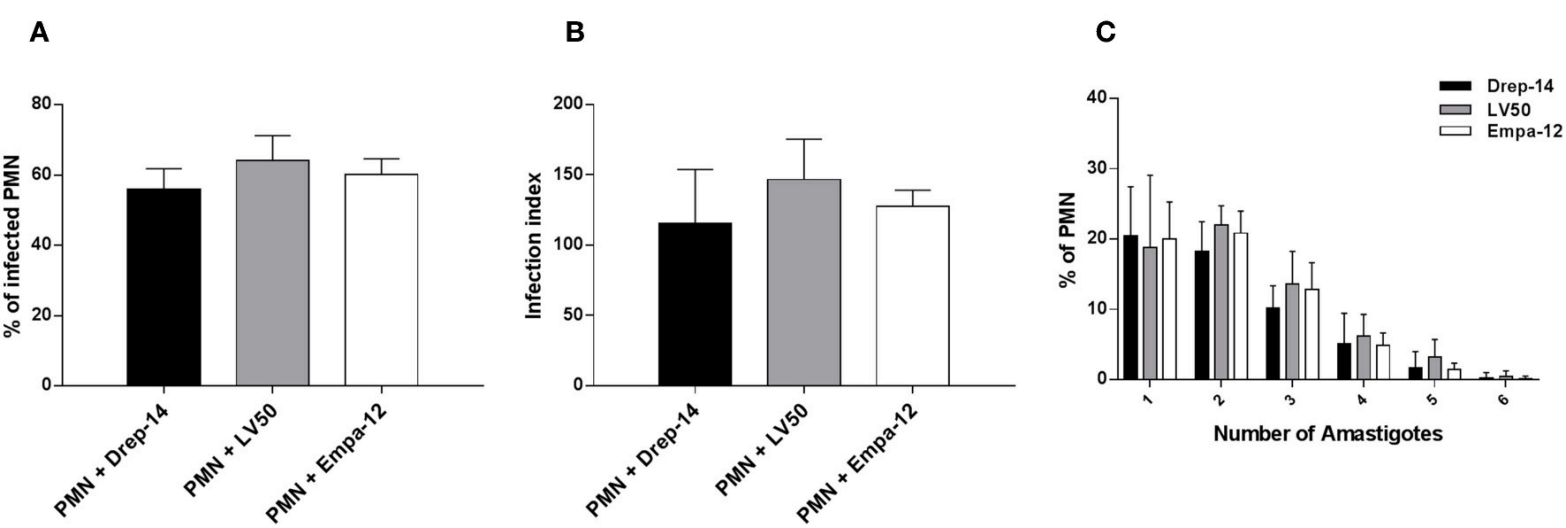
*Leishmania* infection of PMNs. Human neutrophils were infected with *Leishmania* promastigotes (MOI of 10) for 18 h. Then, extracellular parasites were removed and the cells were fixed and stained with May-Grünwald Giemsa kit. **(A)** The percentage of infected PMN, **(B)** the infection index and **(C)** the percentage of cells carrying the designated number of amastigotes were quantified using optical microscopy, by counting at least 100 neutrophils. Data are shown as the mean values from fourteen donors ± standard deviation (SD). Statistical comparisons were performed using the non-parametric Mann-Whitney test.

To investigate the interaction between neutrophils and *Leishmania* promastigotes, 2 × 10^6^ PMN were infected with stationary phase *Leishmania* promastigotes at various MOI (3, 5, 10, and 15) and incubation times (2, 4, 6, 18, and 24 h). The cultures were washed to discard un-internalized parasites. PMN were then fixed and stained with May Grunwald Giemsa and assessed for the percentage of infected cells and parasite burden. The optimum number of infected PMN and intracellular parasites were obtained with 10:1 ratio and after 18 h of incubation (Supplementary Figure 1A). Thus, the experimental conditions at MOI of 10 and 18 h of incubation were chosen for the rest of our study. Neutrophils isolated from 14 healthy donors were infected *ex vivo* with Drep-14, LV50 and Empa-12 strains and infection parameters were determined. Their comparison showed that the neutrophils were similarly infected (Figure 1), according to all parameters tested: the percentage of infection (Figure 1A), the infection index (Figure 1B) and the parasite load (Figure 1C). Intracellular parasites had an amastigote-like morphology and were therefore designated as amastigotes (Supplementary Figure 1B). The percentage of infected neutrophils was in average 56 % [50–68] with Drep-14, 64.2 % [55–72] with LV50, and 60 % [54–67] with Empa-12. The infection index was in average of 115 [88–177] with Drep-14, 146 [129–183] with LV50, and 127 [114–142] with Empa-12. The number of amastigotes within infected cell (Figure 1C) showed that of the infected cells nearly 66 % [63–69] carried only one to two parasites, while approximately 34 % [30–36] of them had multiple parasites (3 to 6 parasites). The mean amastigote number per cell was also similar for each strain, in the range of [2.05–2.27]. Therefore, the mean percentage of infected neutrophils and infection index were similar in neutrophils infected with each strain (*p* > 0.05). Taken together these results demonstrated that in these experimental conditions there were no differences in the ability of the three tested strains to infect human neutrophils.

### *Leishmania* Promastigotes Upregulate Neutrophil Oxidative Burst

**Figure 2:**
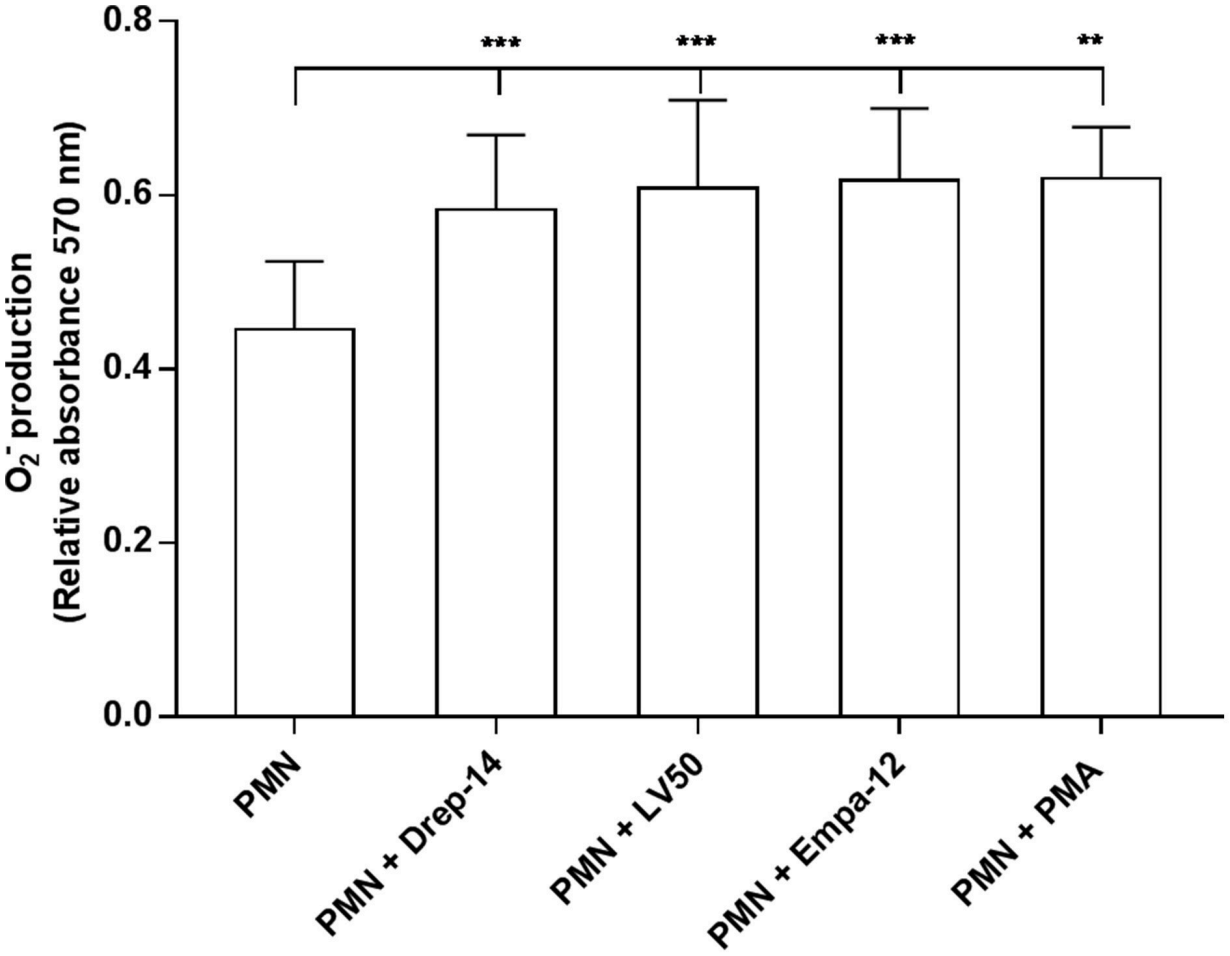
Measures of oxidative burst in *Leishmania* infected neutrophils. PMNs, PMNs exposed to *Leishmania* promastigotes, and PMNs stimulated with 100 nM PMA were incubated in triplicate in 96 well-plates in 100 μL of RPMI-1640/ Glutamax medium plus penicillin (100 U/mL) and streptomycin (100 μg/mL) supplemented with 5 % FBS containing 0.2 % NBT solution to quantify intracellular O_2_^**−**^ production. After 18 h of incubation at 37 °C, in presence of 5 % CO_2_, blue formazan particles were generated after NBT reduction in activated neutrophils. Intracellular formazan deposits were then solubilized by adding 100 μL of 10 % SDS / 0.1 N HCl, and the absorbance of the solution was measured at 570 nm. Data are shown as the mean values of O_2_^**−**^ production from fourteen donors ± SD. Mann-Whitney test was used to compare the absorbance of *Leishmania* infected PMNs to control PMN cultures, and of the infected PMNs in a pair-wise manner; ^**^p < 0.01 and ^***^p < 0.001 indicate statistically significant differences at the indicated *p*-values.

Reactive oxygen species (ROS) are a critical component of the microbicidal activity of neutrophils (Robinson, 2008; Winterbourn et al., 2016). We investigated the effects of infection with the different *Leishmania* strains on superoxide anion (O_2_^**−**^) generation in human neutrophils. We incubated infected and non-infected cells isolated from 14 healthy donors with NBT as described above. As shown in Figure 2, a significant increase in O_2_^**−**^ production was observed following infection with the three *Leishmania* strains or exposure to PMA as compared with PMN alone, whereas no differences could be observed in superoxide anion production by neutrophils infected with the Drep-14, LV50 or Empa-12 strains (*p* > 0.05). These results suggested that the three *Leishmania* strains induced in the same manner ${\text{O}}_{2}^{-}$ production from human neutrophils. To confirm that at 18 h after infection the O_2_^**−**^ production did not reach a plateau, a kinetics of O_2_^**−**^ production (1, 2, 3, 4, 5, 6, and 18 h) was performed for three of the 14 donors. As shown in the Supplementary Figure 2, the tested parasites were able to similarly induce significant O_2_^**−**^ production at all times tested.

### *Leishmania* Promastigotes Trigger Neutrophils Degranulation

**Figure 3:**
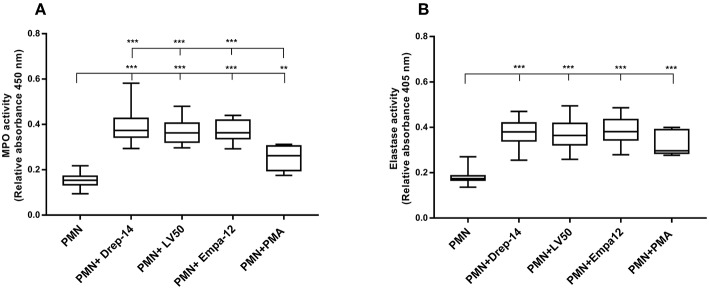
*Leishmania* promastigotes stimulate the degranulation of human PMNs. PMN cultures were infected with stationary phase promastigotes of *Leishmania* strains or incubated with PMA (0 nM & 100 nM, as negative and positive control, respectively) for 18 h, at 37 °C in presence of 5 % CO_2_. Then, supernatants were collected and tested for the presence of enzymes released in the culture medium. **(A)** The enzymatic activity of MPO was quantified by an enzyme-substrate reaction using *o*-Dianisidine and hydrogen peroxide. The absorbance of oxidized *o*-Dianisidine was measured at 450 nm after 10 min of substrate incubation at room temperature. **(B)** The enzymatic activity of neutrophil elastase was determined from the same culture supernatants using a specific synthetic peptide substrate of elastase that is cleaved to colorimetric p-nitroanilide (pNA). The release of pNA was measured at 405 nm after 3 h of incubation at 37 °C. Data are shown as the mean values from fourteen donors ± SD. Mann-Whitney test was used to compare the absorbance of (PMN) vs. each (PMN-*Leishmania* strain) or (PMN-PMA) supernatants, and of the infected PMNs in a pair-wise manner; ^**^p < 0.01 and ^***^p < 0.001 indicate statistically significant differences at the indicated *p*-values.

Degranulation of vesicles into the phagolysosome or in the extracellular space is a key event for microbicidal activity. Contents release of neutrophil granules contributes to the elimination of pathogens (Kumar and Sharma, 2010). To evaluate degranulation, we examined the release of MPO and Neutrophil elastase, which are both azurophilic granule contents, by neutrophils isolated from the 14 healthy donors in the culture medium upon their infection by the different *Leishmania* strains. Enzymatic activities were measured spectrophotometrically in supernatants of non-infected and promastigote-infected PMN through addition of the appropriate substrates (Figure 3). The MPO (Figure 3A) and elastase activities (Figure 3B) were 2.4 and 2 fold higher, respectively, in the supernatants from *Leishmania*- infected neutrophils as compared to the non-infected neutrophils. We also found greater levels of MPO and elastase activity in supernatants from PMA stimulated cells in comparison to untreated neutrophils. Therefore, infection with each of the three strains triggered in the same manner the extracellular release of myeloperoxidase and elastase by human neutrophils. Furthermore, to assess the absence of significant differences in MPO and elastase production between strains within the first hours of infection, a kinetics of released MPO and elastase activities (2, 4, 6, and 18 h) was performed in case of three of the 14 donors. As shown in the Supplementary Figure 3, the three strains significantly, and similarly, increased MPO and elastase release at all-times tested. In conclusion, our data showed that all the tested strains induced in the same way the degranulation of human neutrophils.

### *Leishmania* Promastigotes Induce DNA Release

NETs were described as a host defense mechanism of the innate immune response. NETs involve the release of DNA into the extracellular environment associated with nuclear and granular proteins (Brinkmann et al., 2004; Guimarães-Costa et al., 2009). To determine whether *Leishmania* strains trigger the extracellular release of DNA, neutrophils purified from seven healthy donors were infected or not with parasites for 18 h. Upon this time, extracellular DNA was digested with two restriction enzymes and the DNA released in the supernatants were quantified by fluorometry on a Qubit.

**Figure 4:**
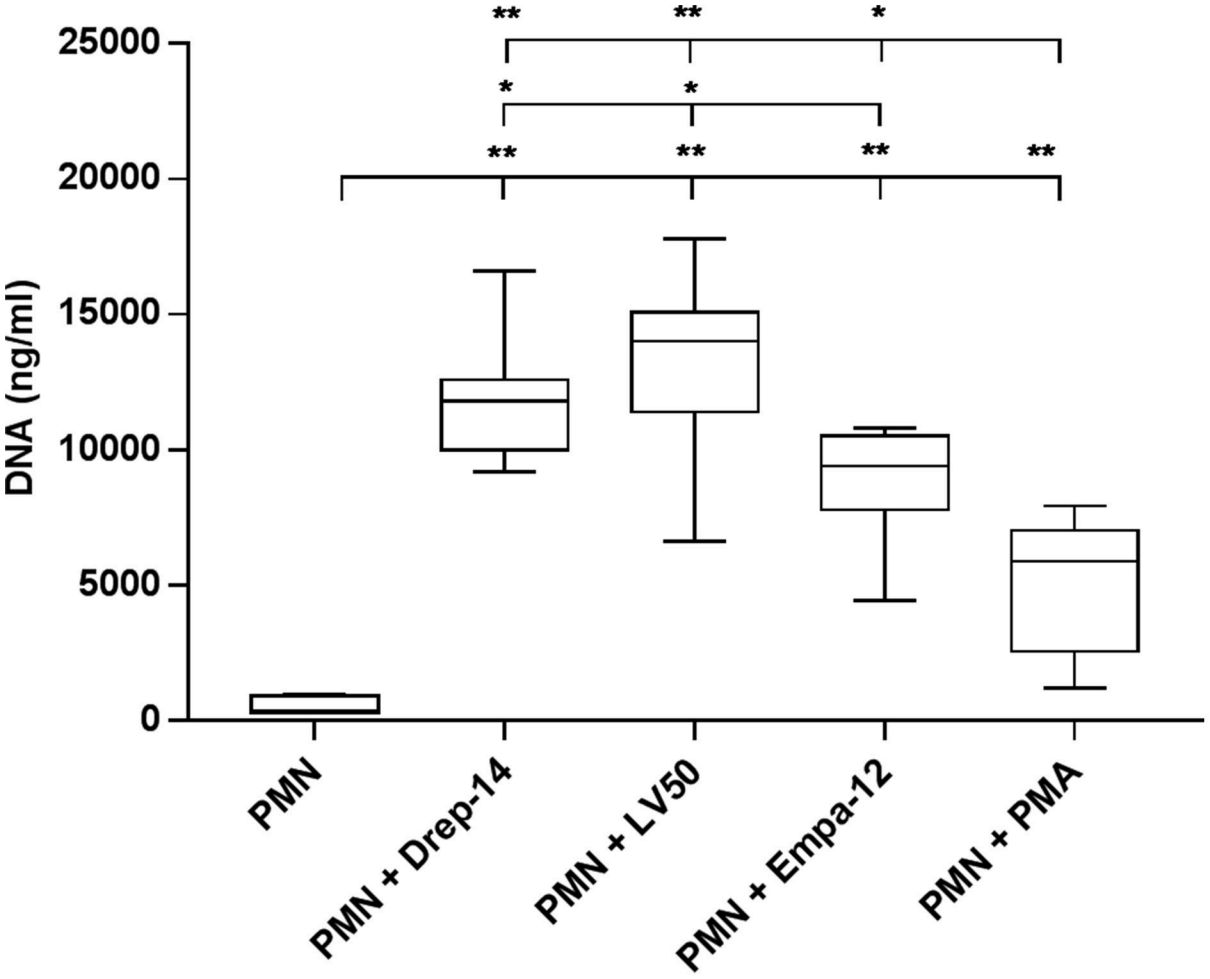
*Leishmania* promastigotes induce DNA release. Neutrophils (2 × 10^5^) were infected with *Leishmania* strains at a 10:1 ratio or with 100 nM PMA as a positive control (0 nM PMA was the negative control) for 18 h at 37 °C in presence of 5 % CO_2_. Then, *EcoR1* and *HindIII* (20 U/mL) were added to the medium to digest the DNA trapped in the released NETs. After 4 h of incubation at 37 °C, the cells were centrifuged and released DNA was quantified in the culture supernatant by DNA quantification using a dsDNA High Sensibility Assay Kit on a Qubit. Data are shown as mean values from seven donors ± SD. Pair wise comparisons of PMN vs. each strain-infected PMNs or vs. PMN-PMA, and of infected PMNs between each other were performed using the Mann–Whitney test; ^*^p < 0.05 and ^**^p < 0.01 indicate statistically significant differences at the indicated *p*-values.

The results showed that all parasite strains significantly induced DNA (and thus likely NETs) release, in higher amounts than non-infected neutrophils and PMA-stimulated neutrophils (*p* < 0.01) (Figure 4). Moreover, the two *L. infantum* strains induced more DNA release from infected neutrophils than the *L. major* strain (*p* < 0.05). Furthermore, to prove the presence of DNA in the supernatants, we did electrophoresis on agarose gels. As shown on Supplementary Figure 4, the supernatants showed the presence of DNA smears ranging from high molecular weight DNA (> 10 Kb) to lower DNA sizes as observed in other studies (Sousa-Rocha et al., 2015; Stephan et al., 2016).

### *Leishmania* Promastigotes Infection Increases Neutrophils Apoptosis Rates

**Figure 5:**
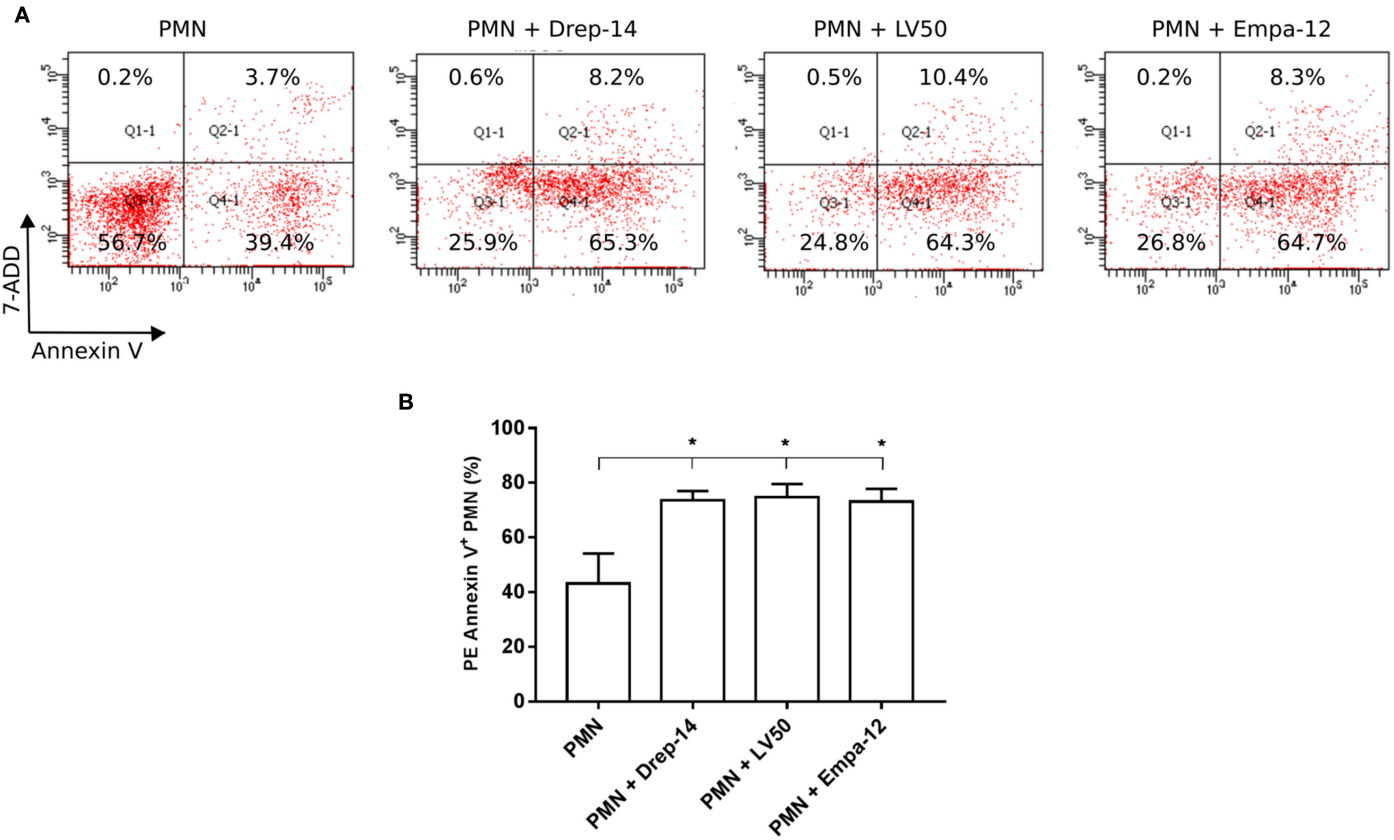
Increased neutrophils apoptosis upon *Leishmania* promastigotes infection. Neutrophils isolated from three donors were infected or not with *Leishmania* parasites (MOI of 10). After 18 h of infection, the cells were labeled with PE Annexin V / 7-ADD and analyzed by FACS. **(A)** Representative dot plots of non-infected PMN and PMN infected with Drep-14 (PMN+Drep-14), with LV50 (PMN+LV50) or with Empa-12 (PMN+Empa-12) were labeled with Annexin V vs. 7-ADD. The plots illustrate the results obtained for one of three donors tested. **(B)** Percentage of PMN Annexin V^+^ of non- infected and infected PMN of three donors. Data are shown as mean percentage of apoptotic PMN ± SD. Pair- wise comparisons of the non-infected PMN vs. each strain- infected PMN were performed using the Mann–Whitney test; ^*^p < 0.05 indicates statistically significant differences.

Neutrophils have a very short life span and they rapidly die via apoptosis. Apoptotic cells express phosphatidylserine (PS), which could be detected by PE Annexin V. To evaluate the effect of *Leishmania* strains on neutrophils apoptosis, neutrophils purified from three healthy donors (among the 14 selected) were infected or not with parasites for 18 h. Then, cells were double stained with Annexin V and the vital dye (7-ADD) to differentiate between viable (PE-Annexin V^−^/ 7-ADD^−^), necrotic (PE-Annexin V^−^/ 7-ADD^+^), early apoptotic (PE-Annexin V^+^/ 7-ADD^−^) and late apoptotic and/or already dead PMN (PE-Annexin V^+^/ 7-ADD^+^). The results showed a significant increase in the percentage of apoptotic cells (PE-Annexin V^+^) following exposure to *Leishmania* strains as compared to PMN alone (Figure 5). Neutrophils viability was significantly decreased in the presence of all tested *Leishmania* strains, whereas we detected low percentages of both necrotic and late apoptotic PMN that were not affected upon *Leishmania* infection (Figure 5A). No statistically significant differences could be observed in apoptosis of neutrophils infected with Drep-14, LV50, or Empa-12 strains (Figure 5B). In conclusion, our data showed that the three *L. infantum* and *L. major* strains tested in our study were able to similarly increase the apoptosis of the tested human neutrophils.

### *Leishmania* Strains Differently Induce Cytokines Release From Neutrophils

**Figure 6:**
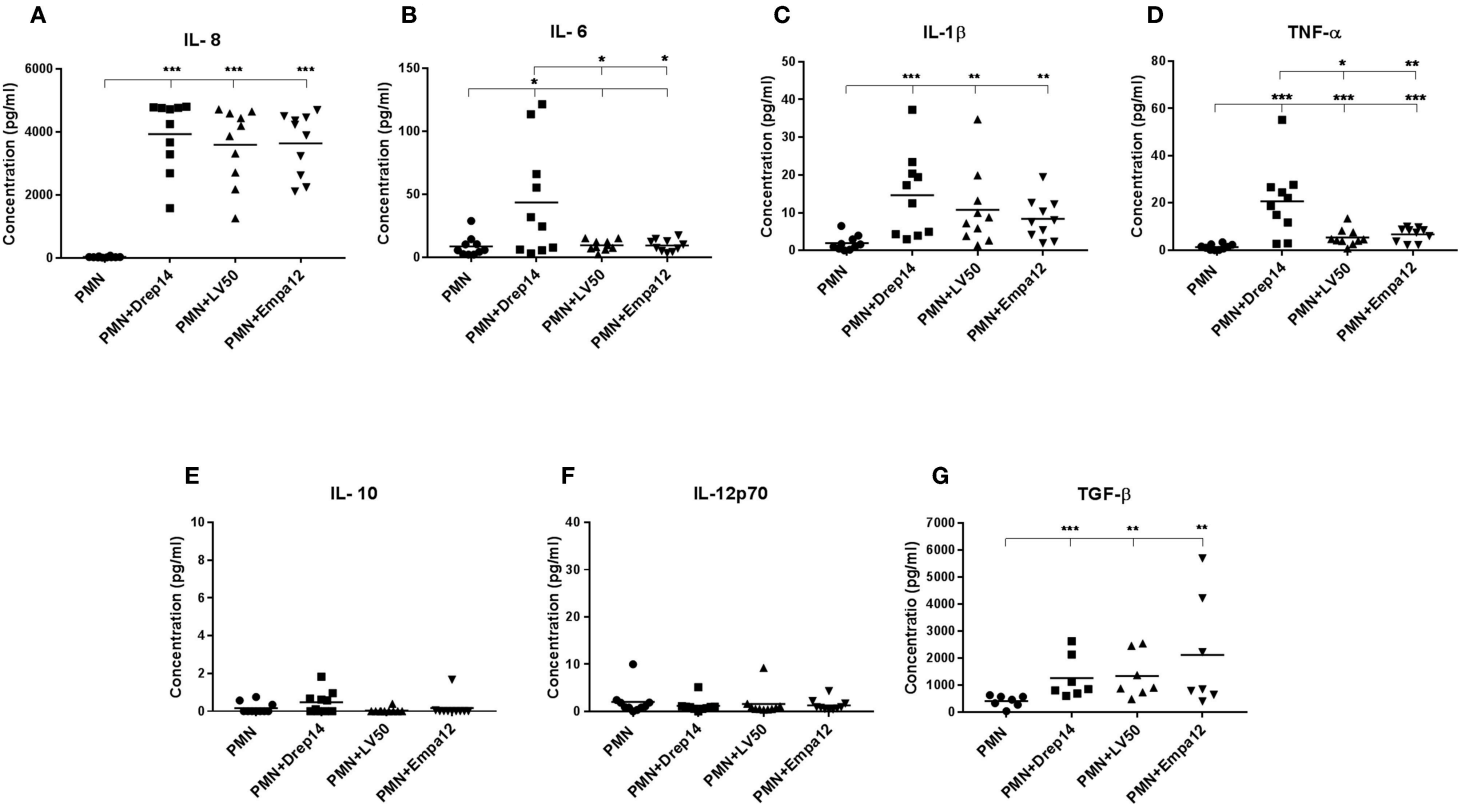
Effect of *Leishmania* strains on cytokines production after *ex vivo* infection of human PMNs. Neutrophils were infected or not by *Leishmania* parasites (MOI of 10). Then, supernatants were collected after 18 h of incubation. The amounts of **(A)** IL-8, **(B)** IL-6, **(C)** IL-1β, **(D)** TNF-α, **(E)** IL-10, and **(F)** IL-12p70 protein were quantified from (50 μL) culture supernatants using the BD Cytometric Bead Array (CBA) Human Inflammation Cytokines kit. The amount of **(G)** TGF-β was quantified from the supernatants (100 μL) by Enzyme-linked immunosorbent assay (ELISA). Results were expressed as the cytokine concentration obtained from seven (TGF-β) or ten sampled donors. The horizontal bars indicate the median value of cytokine production. Statistical pair- wise comparisons of PMN vs. each strain- infected PMN, and of strain- infected PMNs were performed using the Mann–Whitney test; ^*^p < 0.05, ^**^p < 0.01, and ^***^p < 0.001 indicate statistically significant differences at the indicated *p*-values.

As neutrophils are the first cells to arrive at the sites of infection, they may influence the development of the anti-*Leishmania* immune response by producing cytokines and chemokines, which in turn can influence the outcome of disease (Ribeiro-Gomes and Sacks, 2012; Hurrell et al., 2016). In order to evaluate the immune response triggered by the three *Leishmania* strains through the release of cytokines, neutrophils purified from ten healthy donors were incubated in the presence (MOI 10) or absence of each *Leishmania* strain. Supernatants were then collected 18 h after infection, and a cytokine multiplex analysis of culture supernatants was performed by flow cytometry (Figure 6). As shown in Figure 6A, infected neutrophils with each *Leishmania* strain produced approximately 150 fold higher IL-8 amounts than the non-infected neutrophils (Figure 6A). Additionally, IL-1β and (active form) TGF-β production by neutrophils were significantly induced by all strains (Figures 6C,G). Interestingly, we observed that only Drep-14 strain promoted IL-6 release (Figure 6B) by the neutrophils. The production of TNF-α was also significantly induced by all parasite strains (Figure 6D), whereas Drep-14 induced the highest TNF-α amounts as compared to LV50 and Empa-12. The IL-10 and IL-12p70 cytokines (Figures 6E,F) were neither produced in the supernatant of the non-infected nor of the infected neutrophils. All these results demonstrated that the three *Leishmania* strains have differently affected the pattern of cytokines production by the human neutrophils.

### Principal Component Analysis Reveals Strain-Dependent Cytokine Production Profiles

**Figure 7:**
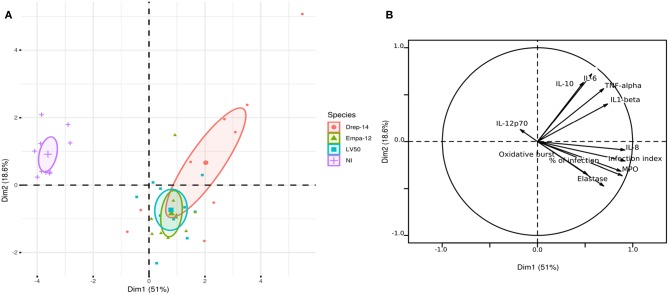
Principal component analysis results. The PCA was performed using data from ten donors for eleven observations (infection index, percentage of infection, Oxidative burst, Elastase, MPO, IL-12p70, IL-8, IL-6, IL-10, IL1-β, and TNF-α) under four conditions (NI = Non-Infected, and three infected conditions with the Drep-14, LV50 or Empa-12 strains). **(A)** The individuals' map shows the distribution of the population projected on the 2D plane composed of the first two principal components PC1 and PC2 (Dim1 and Dim2, respectively). **(B)** The variable factors map shows the projection on the first two principal components (PC1 and PC2) of the different observations.

In order to compare the effect of each donor immune response to the effect of the *Leishmania* strain on the cytokine production profiles by human neutrophils, we generated 3D histograms of the data (Supplementary Figure 5). The histograms suggested no donor-specific variability in cytokines production from one side and a possible strain-specific variability from the other. To statistically confirm such observations, we performed a principal component analysis (PCA) using data from ten individuals (donors) for which 11 observations could be collected under four conditions (non-infected (NI), infected with Drep-14, LV50, or Empa-12 strains). This analysis consisted in an orthogonal linear transformation of the data that led to its projection in a new coordinates system, composed of linearly uncorrelated variables called the principal components (PCs). The objective was to observe a maximum of variance of the data on the first PCs, and thus to be able to reduce the dimension of the data while observing the possible variations among the individuals. Herein, the PCA resulted in 69.6 % of the total variation on the first two principal components (PC1: 51 % and PC2: 18.6 %), which indicated reliability of the analysis. The projection of the individuals on the 2D plot composed by PC1 and PC2, presented a clear separation of the non-infected vs. *Leishmania*-infected individuals (Figure 7A). Observations on all individuals infected by each strain were presented in different colors. The centroids of each strain group and the geometry of the projections suggested that no significant difference could be observed between the LV50 and the Empa-12-infected individuals. Both groups were centered and mostly aggregated in the same plane quarter with a slight diffusion along PC2. Whereas, the Drep14-infected individuals presented a more diffuse geometry along PC1 and PC2, simultaneously. Noticeably, PC1 mostly segregated the non-infected vs. infected populations (Figure 7B) and PC2 distinguished the infection parameters (infection index, percentage of infection, oxidative burst, Elastase, and MPO) vs. the cytokines IL-6, IL-10, IL1-β, and TNF-α production. This suggested that all three strains induced the infection in an equivalent manner, whilst Drep-14 induced different cytokine production profiles as compared to LV50 and Empa-12. IL-8 production presented the least variation between the strains. IL-12p70 production appeared as non-correlated to the infection conditions. Thus, this statistical analysis of all the results together reveals a strain (and species) dependent cytokine production.

### *Leishmania* Strain Dependent Survival Within Infected Neutrophils

**Figure 8:**
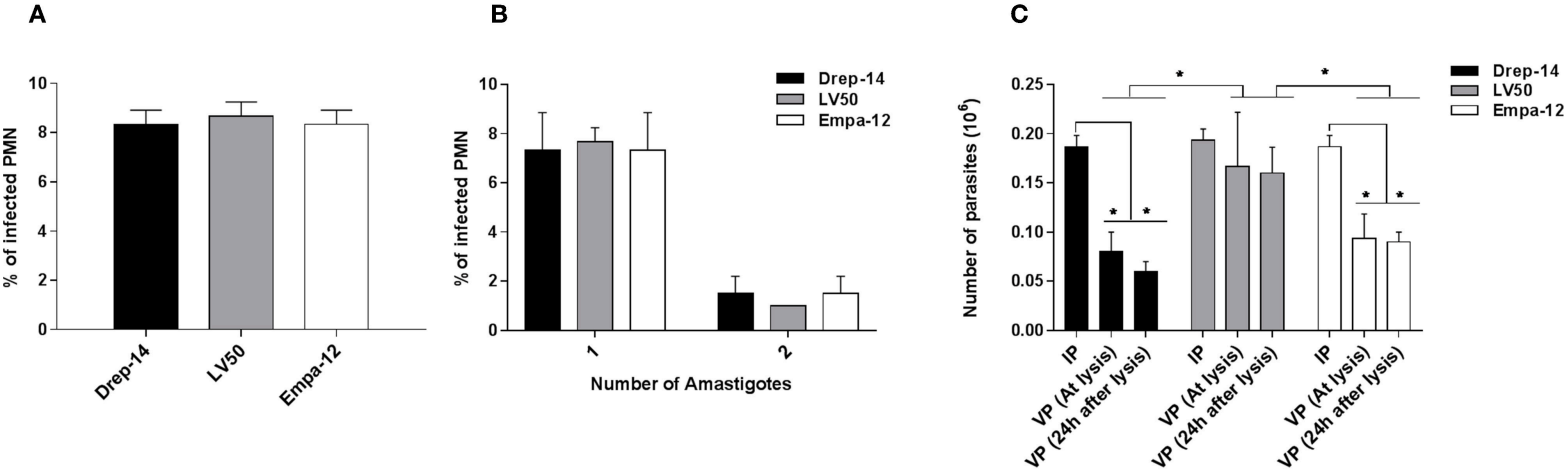
Intracellular *Leishmania* survival in *ex vivo* infected neutrophils. Neutrophils isolated from three donors were infected or not by *Leishmania* parasites (MOI of 0.1) for 18 h. Then, the extracellular parasites were removed and cells were fixed and stained with May-Grünwald Giemsa kit to determine, under optical microscope, the percentage of infected PMN **(A)** and the percentage of cells carrying the designated number of amastigotes **(B)** by counting at least 100 neutrophils. The PMN cultures were lysed at 18 h, by 0.01 % SDS, washed with PBS and resuspended in 100 μL Schneider medium supplemented with 10 % FBS and cultured at 22 °C. **(C)** Parasites viability was assessed right after the lysis of the cells and 24 h later by an MTT assay. Data are shown as mean values of number ± SD. We performed the non-parametric Mann–Whitney test to compare infection parameters **(A,B)**, and in **(C)**: (i) for each strain, the number of Internalized Parasites (IP) within the infected cells vs. the number of Viable Parasites (VP) after lysis at each time point, and (ii) between strains, in a pair- wise manner, the number of surviving parasites at each time point; ^*^p < 0.05 indicates statistically significant differences.

Our previous results indicated that the neutrophils differently responded to the strains tested. So we assessed whether the neutrophils differently impair intracellular parasite survival using a model of infection where most parasites would be internalized, thus leaving very few extracellular ones (Carlsen et al., 2015a). To this end, 2 × 10^6^ neutrophils purified from three donors were infected with 0.2 × 10^6^ parasites (MOI of 0.1) for 18 h and the number of internalized parasites was quantified using optical microscopy. The percentage of infected cells was similar for the 3 strains ≈8 % (Figure 8A). The number of intracellular parasites was estimated to be 0.18 × 10^6^ for the strains Drep-14 and Empa-12, and 0.19 × 10^6^ for LV50 (Figure 8B). These values are considered as equivalent. Thus, all strains had a similar infectivity index in average 28.5 [28–29]. Internalized parasites were then recovered from the infected PMN after cellular lysis, and their viability was assessed right after the lysis and after 24 h of incubation at 22 °C, using an MTT assay. The interpolated number of viable parasites was compared to the intracellular amastigotes counts by microscope and the percentage of viability was determined at the 2 time points (Figure 8C). Whereas, 87.8 and 82 % of LV50 parasites were viable at lysis and 24 h later, respectively, the estimates were significantly reduced in case of the other two strains, Drep-14 (43.6 % vs. 30.4 %) and Empa-12 (48.2 and 48.3 %). The difference in survival of the Drep-14 and EMPA-12 strains *vs*. LV50 was considered to be significant at the two time points tested.

Therefore, while all strains presented the same infectivity, the results clearly indicated that their intracellular survival was different. Noticeably, both dermotropic strains that belong to two different species: *L. infantum* and *L. major* showed poorer survival rates as compared to the viscerotropic LV50 strain (*L. infantum*).

In conclusion, our results suggest that intracellular leishmanicidal ability of the tested human neutrophils will depend on the *Leishmania* strains, even for the same species.

## Discussion

Our objectives were to assess effect of strains of the *L. infantum* and *L. major* species that cause different clinical manifestations (VL or CL) on *ex vivo* human neutrophils. To this end, we have used neutrophils purified from healthy human donors and established conditions that allowed optimal infection of the cells by using stationary phase promastigotes. We reached 65 % infection upon 18 h incubation at an MOI 10. With a higher MOI we observed an extensive cell lysis and so we retained the MOI 10 condition. Previous studies provided evidence that either human or murine neutrophils internalize promastigotes of a range of *Leishmania* species including *L. major* (Laufs et al., 2002; Van Zandbergen et al., 2004; Peters et al., 2008; Mollinedo et al., 2010; Ricci-Azevedo et al., 2016; Ronet et al., 2018) and *L. infantum* (Rousseau et al., 2001; Thalhofer et al., 2011; Marques et al., 2015; Quintela-Carvalho et al., 2017; Sacramento et al., 2017; Valério-Bolas et al., 2019). Species of the *Viannia* subgenus internalized more parasites than those of the *Leishmania* subgenus (*L. infantum*; *L. amazonensis*) (Valério-Bolas et al., 2019). Here we confirmed infection of human neutrophils by *in vitro* promastigote forms of strains of the *L. major* or *L. infantum* species. The intracellular parasites had an amastigote- like morphology and were therefore designated as amastigotes. This indicated that in our experiments the parasites underwent morphological transformation within the neutrophils. Previous studies also observed “amastigotes or amastigote- like forms” within promastigote- infected neutrophils as soon as after 3 h incubation (Marques et al., 2015; Quintela-Carvalho et al., 2017; Valério-Bolas et al., 2019). In addition, we did not observe any significant differences between the strains in the different infection parameters measured at two different MOIs (10 and 0.1). Interestingly, with the MOI 10 experiments, 34 % of the infected cells were found to contain 3 to 6 amastigotes. This may be correlated with the fact that more than 2 parasites were uptaken at this higher MOI. Amastigotes were also seen to replicate within neutrophils (Hurrell et al., 2017) but we cannot ascertain it was the case here.

Neutrophils, as key component of the innate immune system, play a pivotal role in first line defense against invading pathogens through phagocytosis, the release of granule contents (Segal, 2005; Nauseef, 2007) and the production of NETs (Brinkmann et al., 2004; Guimarães-Costa et al., 2009). Upon phagocytosis, neutrophils NADPH oxidase is activated and produces the superoxide anion (O_2_^**−**^) (Pham, 2006) leading to the production of antimicrobial molecules such as ROS, which contribute to the killing of intracellular parasites such as *L. donovani* and *L. major* (Pearson and Steigbigel, 1981; Laufs et al., 2002). Neutrophils from VL patients displayed impaired effector functions but they were able to phagocyte *L. donovani* similarly to neutrophils from healthy controls, which suggested a role in the survival and dissemination of *L. donovani* (Yizengaw et al., 2016). In contrast, other studies documented differences in the induction of oxidative burst response depending on *Leishmania* species. It was significantly higher in *L. braziliensis*-infected murine neutrophils than in those infected by *L. amazonensis* (Carlsen et al., 2015a). Herein, we report that the tested *L. infantum* and *L. major* strains similarly induced high oxidative burst in the infected human neutrophils at different time points.

Release of azurophilic granules that contain antimicrobial proteins such as NE and MPO, into the phagolysosome or the extracellular space of infected neutrophils, plays a crucial role in pathogens elimination (Segal, 2005; Nauseef, 2007). Degranulation was observed in human, murine and in canine neutrophils infected by different *Leishmania* species: *L. braziliensis* (Carlsen et al., 2015a; Falcão et al., 2015), *L. amazonensis* (Tavares et al., 2014; Carlsen et al., 2015a), *L. infantum* (Marques et al., 2015; Pereira et al., 2017). Here, we have observed that the tested *L. infantum* and *L. major* strains induced the neutrophils degranulation with similar NE and MPO production.

The release of NETs, also known as NETosis, in response to different *Leishmania* species has been described in human or murine neutrophils. These extracellular structures made of fiber-, web- and tube- like elements emitted by activated neutrophils are rich in histones (toxic proteins to pathogens), DNA, and granular and cytosolic proteins such as NE or MPO, and are able to entrap the parasites (Guimarães-Costa et al., 2009; Valério-Bolas et al., 2019). In case of *L. infantum*, it appeared that tube-like structures could allow coiling phagocytosis of promastigotes by murine neutrophils (Valério-Bolas et al., 2019), an unconventional phagocytosis mechanism also used by macrophages to internalize the parasites (Hsiao et al., 2011). *L. amazonensis* induced the formation of NETs by a mechanism involving surface lipophosphoglycan (LPG) and was killed by them (Guimarães-Costa et al., 2009). *L. donovani* and *L. infantum* were also shown to induce NETs release but they escaped NETs killing owing to their LPG (Gabriel et al., 2010) or their 3'-nucleotidase/nuclease activity (Guimarães-Costa et al., 2014), respectively. Furthermore, *L. mexicana* induced the formation of NETs but was also not killed by them through a not reported mechanism (Hurrell et al., 2015). We here report that the 3 *L. infantum* and *L. major* strains induced the release of DNA in the extracellular medium by human neutrophils, suggesting the presence of NETs. *L. infantum* released more DNA amounts than *L. major* suggesting that the intensity of this release may be species-specific. A difference between the DNA amounts released by the two *L. infantum* strains was also noticed although not considered significant. In line with this observation, it was recently shown that murine neutrophils exposed to *L. amazonensis* emitted fewer NETs than those exposed to *L. shawi* or *L. guyanensis* (Valério-Bolas et al., 2019). *L. infantum* also induced more NETs release than *L. major* in human neutrophils (Guimarães-Costa et al., 2009).

Different studies reported that *L. major, L. donovani* and *L. infantum* can delay human, murine or canine neutrophils apoptosis and prolong the cell's life span to ensure an intracellular environment favorable to parasites survival, and their silent entry in macrophages (Aga et al., 2002; Gueirard et al., 2008; Sarkar et al., 2013; Marques et al., 2015; Pereira et al., 2017). In contrast, *L. amazonensis* and *L. braziliensis* induced murine neutrophils apoptosis and accelerated their death (Carlsen et al., 2013; Falcão et al., 2015). In the present study the tested *L. infantum* and *L. major* strains increased apoptosis in the donors tested. This difference between our results and those reported in previous studies for these species could be due to the difference between strains, the genetic background of the donors and/or the experimental conditions (MOI, infection time, etc). Further studies are necessary to further investigate apoptosis induced by these species and cellular mechanisms involved in death. Notably *L. infantum* infected neutrophils were shown to undergo necroptosis in presence of specific caspase 8 inhibitor (and so when apoptosis was inhibited) (Barbosa et al., 2018).

In addition to the classical functions of phagocytosis and killing of invading pathogens, neutrophils can modulate the immune responses against *Leishmania* infection by secreting chemokines that attract macrophages and dendritic cells to the site of infection (Ribeiro-Gomes and Sacks, 2012; Hurrell et al., 2016), and also cytokines that influence T cells differentiation (Tacchini-Cottier et al., 2000). Indeed, the early wave of neutrophils in *L. major*-infected BALB/c was shown to express IL-4, and to induce the development of a Th2 response and the partial control of the disease (Tacchini-Cottier et al., 2000) or its exacerbation (Chen et al., 2005). Furthermore, the interactions between neutrophils and macrophages or dendritic cells were shown to influence the outcome of *L. major* infection in animal models (Ribeiro-Gomes et al., 2004, 2012). To our knowledge, the present study is the first addressing the determination of cytokines produced by human neutrophils exposed to different *Leishmania* species using cytometry bead assays (CBA). The choice of this method rather than more classical ones: ELISA or real time PCR (RT-PCR), relied on the limited sample volume needed for the CBA assays, their high sensitivity, the time gain and the diminution of inter assay variations (Faresjö, 2014).

IL-8 enhances the early recruitment of neutrophils to the site of infection (Müller et al., 2001) and activates their functions, such as the phagocytosis (Scapini et al., 2000). IL-8 produced by neutrophils seems to have a limited role in human leishmaniasis as neutrophils from either asymptomatic or non-healing individuals produced high and similar levels of IL-8 after exposure to *L. major* (Safaiyan et al., 2011). Here, we report that human neutrophils exposed to *L. infantum* or *L. major* also produced high amount of IL-8 as in previously reported studies (Laufs et al., 2002; Van Zandbergen et al., 2002; Safaiyan et al., 2011; Keyhani et al., 2014).

TNF-α produced by neutrophils plays an important role for leucocytes migration and for DC and macrophages activation and differentiation (Nathan, 2006). Furthermore, it can induce the neutrophils degranulation at the site of infection, thus it influences the development of an efficient immune response against the parasite (Nathan, 2006). Our results showed that all *Leishmania* strains tested induced the production of TNF-α by the neutrophils of the healthy donors of this study as previously described in case of *L. major* exposed human neutrophils (Safaiyan et al., 2011), or *L. braziliensis*- exposed murine neutrophils (Falcão et al., 2015). Notably, TNF-α release was significantly higher from Drep-14- infected neutrophils than from LV50 and Empa-12- infected ones.

IL-12 and IL-10, which are important immune modulatory cytokines known to favor Th1 or Th2 type responses, respectively, were not induced by the strains tested as reported in previous studies using human and murine neutrophils (Van Zandbergen et al., 2006; Falcão et al., 2015). In contrast, neutrophils from *L. major* infected C57BL6 mice were able to induce the secretion of IL-12p70 and IL-10 (Charmoy et al., 2007). This discrepancy could be due to the host origin of the neutrophils. In line with this hypothesis, in the same study, *L. major*-infected BALB/c neutrophils were unable to induce the secretion of IL-12p70 and IL-10 (Charmoy et al., 2007).

The production of anti-inflammatory TGF-β was associated to the development of non-protective response against *L. major* infection (Van Zandbergen et al., 2006). Furthermore, it was shown that TGF-β favors the uptake of apoptotic cells by macrophages (Fadok et al., 1998). Our observation that human neutrophils produced high amounts of TGF-β after exposure to *L. infantum* or *L. major* strains is consistent with previous studies that highlighted the prominent role of neutrophils in the creation of a microenvironment favorable for parasite survival and the exacerbation of the disease (Safaiyan et al., 2011; Hurrell et al., 2015). In contrast to our results and other studies, Keyhani et al. reported that *L. major* failed to induce expression of TGF-β mRNA in human neutrophils (Keyhani et al., 2014). This discrepancy could be due to the methods used for quantification of cytokine expression: CBA in our study and RT-PCR in the mentioned one, and the other experimental conditions.

IL-1β is a pro-inflammatory cytokine that has a controversial role in murine leishmaniasis. IL-1β promotes the development of leishmaniasis in *L. major* infected susceptible BALB/c mice (Voronov et al., 2010). Furthermore, the inflammasome -derived IL-1β production was important to develop an efficient immune response against *L. braziliensis, L. amazonensis*, and *L. chagasi* infection (Lima-Junior et al., 2013) but it failed to induce resistance against the infection of the resistant C57BL/6 mice by *L. major* Seidman strain (*Lm*Sd) (Charmoy et al., 2016) or the hamster infection by *L. donovani* (Dey et al., 2018). Both studies highlighted the role of the early production of IL-1β in sustaining the recruitment of neutrophils and in inducing the exacerbation of the disease (Charmoy et al., 2016; Dey et al., 2018). Few studies assessed the production of IL-1β by human neutrophils. Our results showed that human neutrophils exposed to *L. infantum* or to *L. major* were able to produce IL-1β as previously described (Keyhani et al., 2014). Contrary to our results, it was also shown that *L. infantum* down regulates the expression of IL-1β in murine neutrophils (Marques et al., 2015). This discrepancy could be due to the strains, the host origin of the neutrophils, or the methods used for the detection of this cytokine: CBA detection of the protein vs. quantitative measure of the transcript, or to the time of detection: 18 h here and 3 h in the mentioned study.

IL-6 is a pleiotropic cytokine produced by many cells that is involved in B cell maturation, macrophages differentiation and promotion of Th2 differentiation (Kishimoto, 2005; Kopf et al., 2010). In addition, with TGF-β, IL-6 induces the differentiation of Th17 cells and inhibits regulatory T cells generation (Bettelli et al., 2006; Kimura and Kishimoto, 2010). IL-6 plays also a pivotal role in the regulation of neutrophils trafficking during inflammation, by regulating the production of chemokines and by inducing neutrophils apoptosis (Fielding et al., 2008). The role of IL-6 in leishmaniases has been assessed in animal models. It was shown that BALB.B mice that are deficient for IL-6 were still susceptible to *L. major* infection, and they were not able to resolve their infection (Titus et al., 2001). In contrast, IL-6 deficient C57BL/6 mice were able to control the infection by *L. major* as the wild type corresponding mice (Moskowitz et al., 1997). Furthermore, multiple studies have shown a correlation between the high level of some cytokines, including IL-6, and the severity of visceral leishmaniasis (Ansari et al., 2006; Van Den Bogaart et al., 2014; Dos Santos et al., 2016; Ramos et al., 2016) and cutaneous leishmaniasis (Latifynia et al., 2012; Espir et al., 2014). To our knowledge, this is the first study addressing the determination of IL-6 protein levels produced by human neutrophils after exposure to *Leishmania* species. Our results showed that only the Drep-14 strain induced the production of IL-6 by human neutrophils. Notably, this strain also induced higher levels of TNF-α and IL-1β than the other two strains, LV50 and Empa-12 while the levels were comparable in case of the 4 other cytokines measured.

Collectively, our results suggest that the response of human neutrophils *in vitro*, at least for the production of cytokines and DNA release, seem to depend on the *Leishmania* strains and species. Although, the three *Leishmania* strains used in the present study induced very high and comparable levels of TGF-β, there was a differential production of the other inflammatory cytokines: TNF-α, IL-1β, and IL-6 according to the strain and species. Importantly, the statistical analysis of our results consolidated the fact that the strain (within the same species) appeared as the main drive in influencing the donors' cell response to the infection. This is further corroborated by the measures of parasite survival. In our experiment, while all strains presented similar infection index, the number of viable parasites was significantly lower in case of the dermotropic strains Drep-14 and Empa-12 that belong to two different species, *L. infantum* and *L. major*. Whereas, in case of the viscerotropic *L. infantum* (LV50 strain) the number of rescued parasites was similar to the number of internalized ones. This indicates that different leishmanicidal mechanisms are may be triggered within the infected cells according to the parasite strains and species. Importantly, our results point that in the interaction of a *Leishmania* species with human blood neutrophils, the parasite strains may differently influence the fate of the infection. Taking account that only three laboratory strains of two species were used in the present study, it would be interesting to assess the effect of more strains and clinical isolates of these species on human neutrophils infection and activation. Interestingly, this would also open ways to study relationship between clinical origin or genetic background of the strains (/species) and the immune response induced.

In conclusion, the present study established an infection model of human neutrophils to evaluate *ex vivo* their responses to *L. infantum* or *L. major* strains infection. Our study clearly demonstrated that the strains were able to induce similar *ex vivo* activation and apoptosis of the tested human neutrophils. However, they differently triggered DNA and inflammatory cytokines release from the neutrophils suggesting that these responses are *Leishmania* species- and strain- specific notably in case of *L. infantum*. Intracellular survival of the parasites also depended on the strains and species. Further study on the mechanisms involved in the responses triggered by these strains needs to be developed. Likewise, the effect of these differentially activated neutrophils and different parasite survival on macrophage infection needs investigation.

## Ethics Statement

This study was carried out in accordance with the recommendations of The Ethical committee of the Institut Pasteur de Tunis with written informed consent from all subjects. All subjects gave written informed consent in accordance with the Declaration of Helsinki. The protocol was approved by The Ethical committee of the Institut Pasteur de Tunis.

## Author Contributions

IG, MB, and RO conceived and designed the experiments. RO performed the experiments. RO and SM performed the ELISA experiment. RO and EH-S performed the statistical analysis. RO, MB, SM, and MBA performed the FACS experiments. RO, MB, EH-S, MBA, and IG analyzed the data. RO, EH-S, IG, and MB drafted the manuscript. All authors read, edited, and approved the final manuscript.

### Conflict of Interest Statement

The authors declare that the research was conducted in the absence of any commercial or financial relationships that could be construed as a potential conflict of interest.
