# Supplementary material for: Infection of Human Neutrophils With Leishmania infantum or Leishmania major Strains Triggers Activation and Differential Cytokines Release
Source: Front Cell Infect Microbiol. 2019 May 10;9:153. doi: 10.3389/fcimb.2019.00153 (PMC6524560; doi:10.3389/fcimb.2019.00153)
Supplement: Supplementary file 1 [file Data_Sheet_1.pdf]

(A)

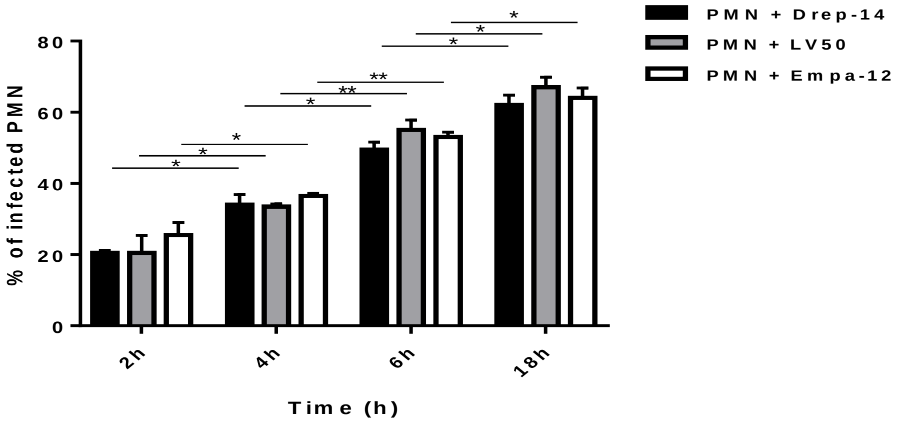

(B)

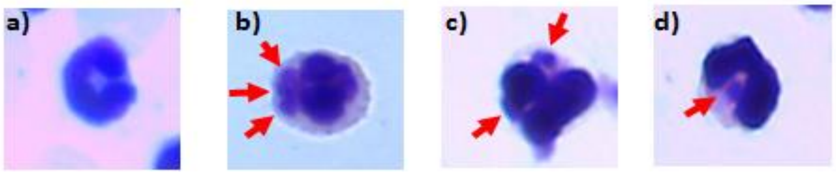

Supplementary Figure 1. *Leishmania* strains infection of PMNs. Human neutrophils were infected or not with stationary phase *Leishmania* promastigotes (MOI of 10) at 37 °C and 5 % CO_2 in RPMI-1640/ Glutamax medium containing penicillin (100 U/mL) and streptomycin (100 µg/mL) supplemented with 5 % FBS. Then, extracellular parasites were removed and the cells were fixed and stained using a May-Grünwald Giemsa staining kit. **(A) Kinetics of the infection.** The percentage of infected neutrophils at various time points (2 h, 4 h, 6 h and 18 h) of infection, were quantified using optical microscopy. Data are shown as the mean values from three donors ± standard deviation (SD). Statistical comparisons were performed using the non parametric Mann-Whitney test; * ($p < 0.05$) and ** ($p < 0.01$) indicate statistically significant differences at the indicated *p* values. **(B) Intracellular amastigotes within the infected neutrophils.** Intracellular parasites having an amastigote- like morphology, therefore designated as amastigotes, can be observed in the infected PMN (× 1000 magnification). **a)** Non- infected PMN; **b)** Drep-14 infected PMN; **c)** LV50 infected PMN, **d)** EMPA-12 infected PMN. The arrows point to the amastigote forms.
