# Supplementary material for: Infection of Human Neutrophils With Leishmania infantum or Leishmania major Strains Triggers Activation and Differential Cytokines Release
Source: Front Cell Infect Microbiol. 2019 May 10;9:153. doi: 10.3389/fcimb.2019.00153 (PMC6524560; doi:10.3389/fcimb.2019.00153)
Supplement: Supplementary file 2 [file Data_Sheet_2.PDF]

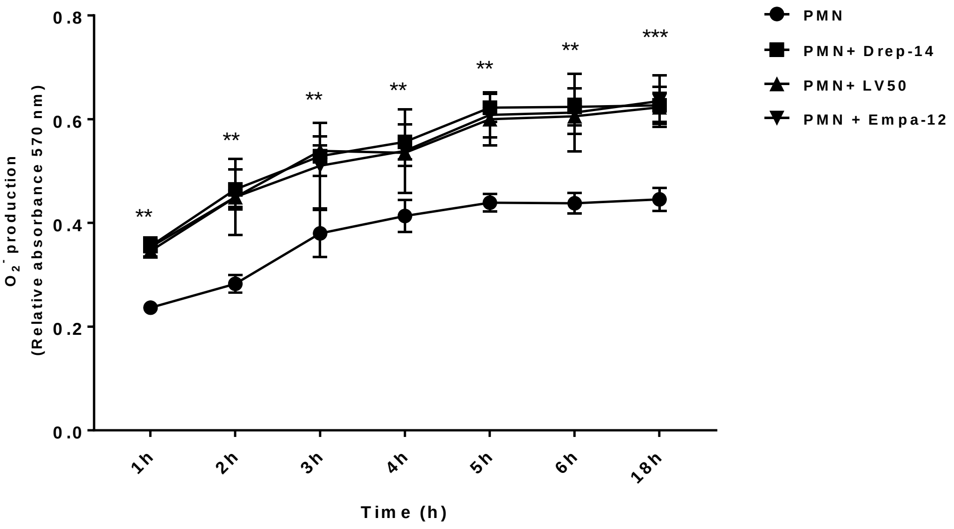

Supplementary Figure 2. **Kinetics of O_2^- production**. PMNs and PMNs exposed to *Leishmania* promastigotes (MOI: 10) were incubated in triplicate in 96 well plates in 100 µL of RPMI-1640/ Glutamax medium plus penicillin (100 U/mL) and streptomycin (100 µg/mL) supplemented with 5% FBS containing 0.2% NBT solution to quantify intracellular O_2^- production. After 1h, 2h, 3h, 4h, 5h, 6h and 18 h of incubation at 37°C, in presence of 5% CO_2, blue formazan particles were generated after NBT reduction in activated neutrophils. Data are shown as the mean values of technical replicates from three donors ± SD. Mann-Whitney test was used to compare the absorbance of (PMN) *vs.* each (PMN-*Leishmania* strain) supernatants, and of the infected PMNs in a pair-wise manner, ** ($p < 0.01$) and *** ($p < 0.001$) indicate statistically significant differences at the indicated p values. There is a significant production of O_2^- at all time points with no difference between the strains.
