# Supplementary material for: Infection of Human Neutrophils With Leishmania infantum or Leishmania major Strains Triggers Activation and Differential Cytokines Release
Source: Front Cell Infect Microbiol. 2019 May 10;9:153. doi: 10.3389/fcimb.2019.00153 (PMC6524560; doi:10.3389/fcimb.2019.00153)
Supplement: Supplementary file 3 [file Data_Sheet_3.PDF]

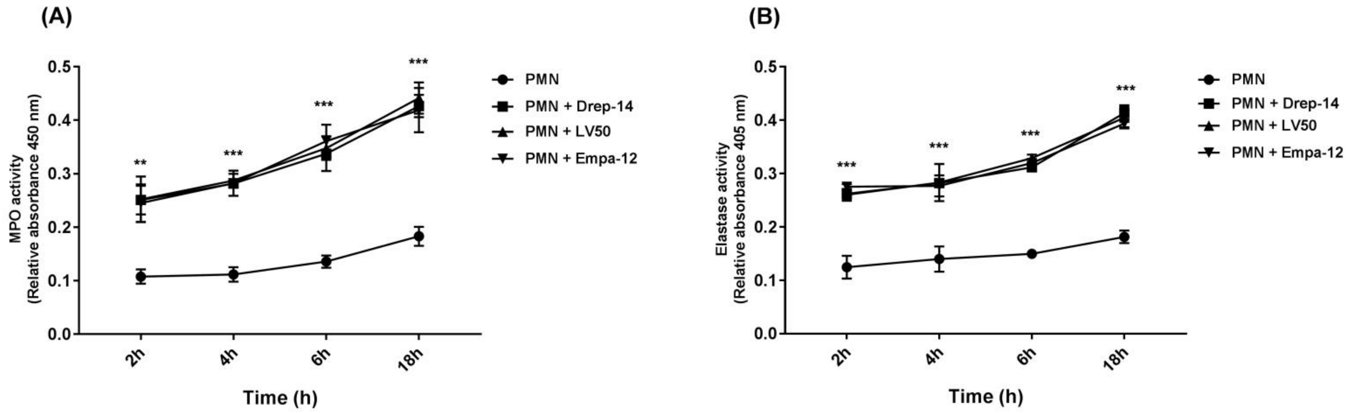

Supplementary Figure 3. **Kinetics of released MPO and elastase activities**. PMN cultures were infected with stationary phase promastigotes of *Leishmania* strains (MOI: 10). Then, supernatants were collected after 2h, 4h, 6h and 18h of incubation and tested for the presence of enzymes released in the culture medium. **(A) The enzymatic activity of MPO** was quantified by an enzyme-substrate reaction using *o*-Dianisidine and hydrogen peroxide. **(B) The enzymatic activity of neutrophil elastase** was determined from the same culture supernatants using a specific synthetic peptide substrate of elastase. Data are shown as the mean values of technical replicates from three donors ± SD. Mann-Whitney test was used to compare the absorbance of (PMN) *vs.* each (PMN-*Leishmania* strain) supernatants, and of the infected PMNs in a pair-wise manner, ** ($p < 0.01$) and *** ($p < 0.001$) indicate statistically significant differences at the indicated p values.
