# Supplementary material for: Infection of Human Neutrophils With Leishmania infantum or Leishmania major Strains Triggers Activation and Differential Cytokines Release
Source: Front Cell Infect Microbiol. 2019 May 10;9:153. doi: 10.3389/fcimb.2019.00153 (PMC6524560; doi:10.3389/fcimb.2019.00153)
Supplement: Supplementary file 4 [file Data_Sheet_4.PDF]

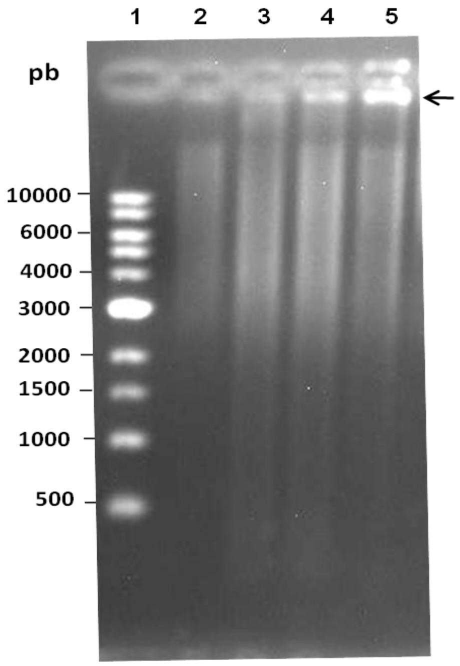

Supplementary Figure 4. A representative illustration of extracellular DNA digestion profiles. Neutrophils (2 × 10^6) were infected with *Leishmania* strains at a 10:1 ratio for 18 h. Then, *EcoR1* and *Hind III* (20 U/mL) were added to the media to digest the DNA trapped in the released NETs. After 4 h of incubation at 37°C, the cells were centrifuged and the supernatants were collected. The presence of DNA and its profile were evaluated by 0.7% agarose gel electrophoresis and staining with Ethidium Bromide (0.5 µg/mL). In infected cells, the smears extend from high molecular weight (> 10Kb) to below 500 pb. The arrow indicates very high MW DNA trapped close to the wells. Lane1: 1Kb DNA ladder, lane 2: supernatant of non- infected PMN, lane 3: supernatant of Drep-14 infected PMN, lane 4: supernatant of LV50 infected PMN and lane 5: supernatant of Empa-12 infected PMN.
