# Supplementary material for: Infection of Human Neutrophils With Leishmania infantum or Leishmania major Strains Triggers Activation and Differential Cytokines Release
Source: Front Cell Infect Microbiol. 2019 May 10;9:153. doi: 10.3389/fcimb.2019.00153 (PMC6524560; doi:10.3389/fcimb.2019.00153)
Supplement: Supplementary file 5 [file Data_Sheet_5.pdf]

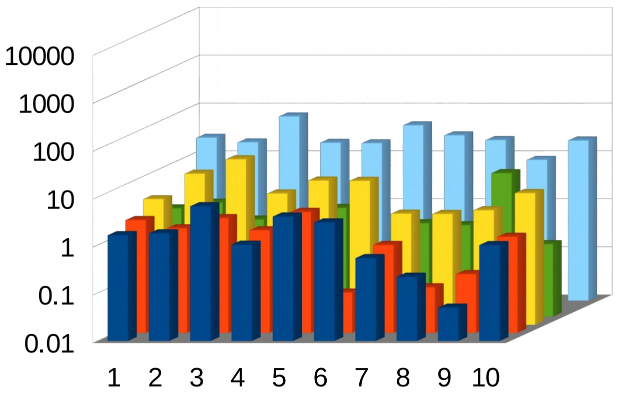
10000
1000
100
10
1
0.1
0.01
1 2 3 4 5 6 7 8 9 10

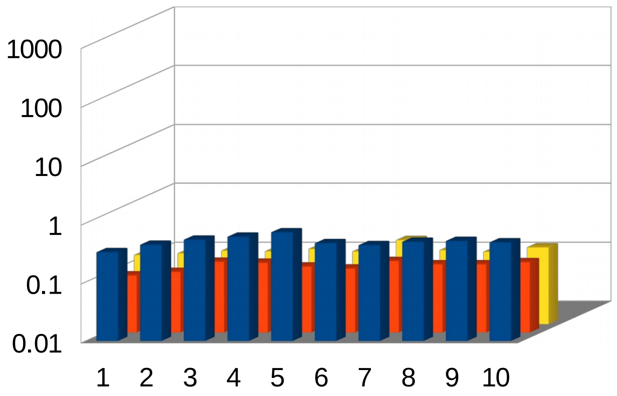
1000
100
10
1
0.1
0.01
1 2 3 4 5 6 7 8 9 10

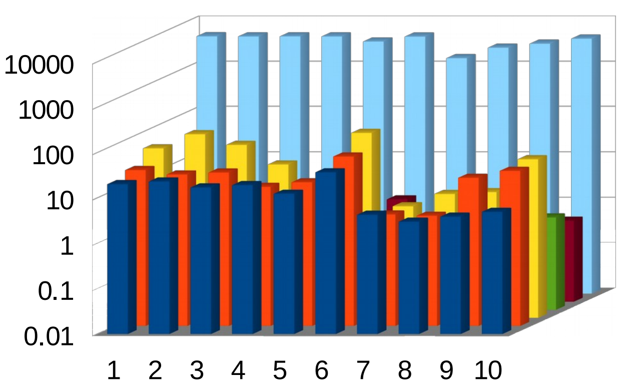
10000
1000
100
10
1
0.1
0.01
1 2 3 4 5 6 7 8 9 10

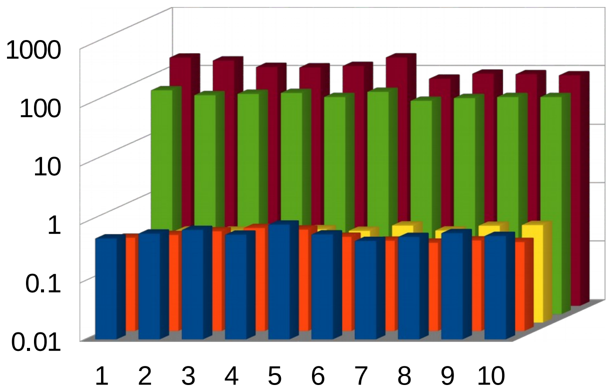
1000
100
10
1
0.1
0.01
1 2 3 4 5 6 7 8 9 10

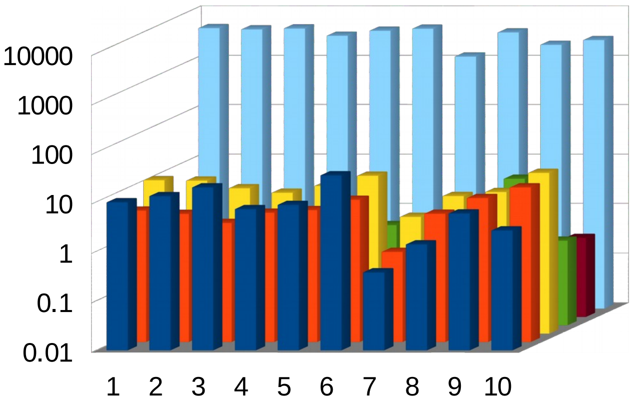
10000
1000
100
10
1
0.1
0.01
1 2 3 4 5 6 7 8 9 10

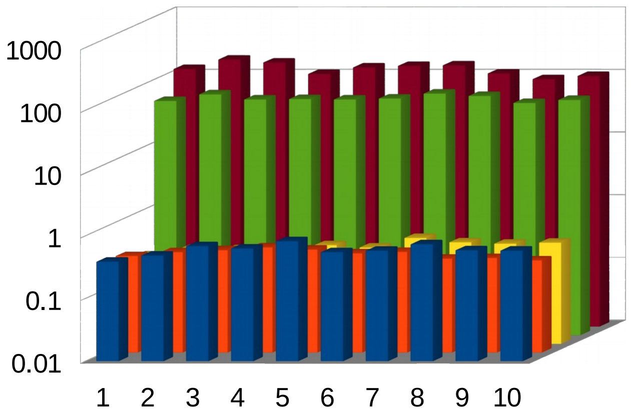
1000
100
10
1
0.1
0.01
1 2 3 4 5 6 7 8 9 10

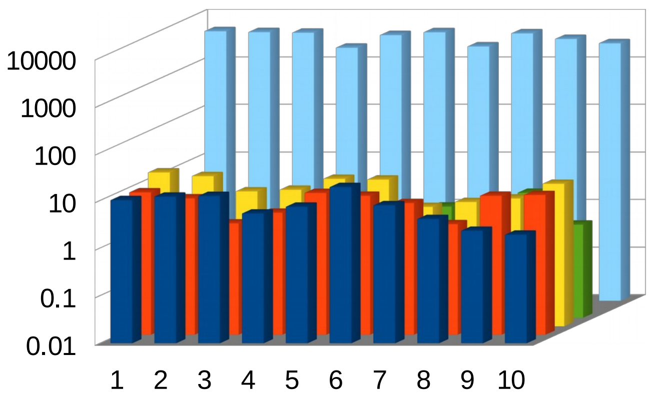
10000
1000
100
10
1
0.1
0.01
1 2 3 4 5 6 7 8 9 10

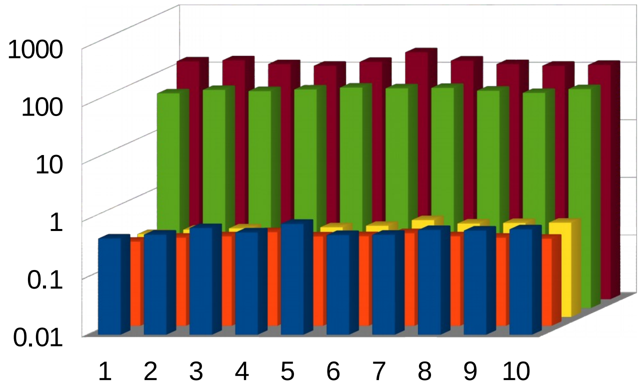
1000
100
10
1
0.1
0.01
1 2 3 4 5 6 7 8 9 10

Supplementary Figure 5. A 3D representation of the variation of the measured observations. The first column contains data related to cytokine induction. The second column contains data related to the infection parameters. The first line contains data related to the non infected (NI) condition. The second line contains data of the Drep-14-related infection. The third line contains data of the LV50-related infection. The fourth line contains data of the EMPA-12-related infection. A logarithmic scale was used for the Y-axis in order to overcome the skewness of the data (very large and very small values within its distribution).
