# Supplementary material for: Infection of Human Neutrophils With Leishmania infantum or Leishmania major Strains Triggers Activation and Differential Cytokines Release
Source: Front Cell Infect Microbiol. 2019 May 10;9:153. doi: 10.3389/fcimb.2019.00153 (PMC6524560; doi:10.3389/fcimb.2019.00153)
Supplement: Supplementary file 6 [file Data_Sheet_6.PDF]

Supplementary Table. 1. Contribution of each donor to the experiments

Test / Donors	Infect Rate	Infect Index	Burst	MPO	Elastase	IL-1β	TNF-α	IL-6	IL-10	IL-12	IL-8	TGF-β	DNA release	Apoptosis	Intracellular parasites survival	Kinetics of Infection (MOI 10)	Kinetics of O_2^-, MPO & NE
**1**	**X**	**X**	**X**	**X**	**X**	**X**	**X**	**X**	**X**	**X**	**X**	X	X	X	X	X	X
**2**	**X**	**X**	**X**	**X**	**X**	**X**	**X**	**X**	**X**	**X**	**X**	X	X				
**3**	**X**	**X**	**X**	**X**	**X**	**X**	**X**	**X**	**X**	**X**	**X**		X				
**4**	**X**	**X**	**X**	**X**	**X**	**X**	**X**	**X**	**X**	**X**	**X**	X	X				
**5**	**X**	**X**	**X**	**X**	**X**	**X**	**X**	**X**	**X**	**X**	**X**	X	X	X	X	X	X
**6**	**X**	**X**	**X**	**X**	**X**	**X**	**X**	**X**	**X**	**X**	**X**	X	X				
**7**	**X**	**X**	**X**	**X**	**X**	**X**	**X**	**X**	**X**	**X**	**X**						
**8**	**X**	**X**	**X**	**X**	**X**	**X**	**X**	**X**	**X**	**X**	**X**						
**9**	**X**	**X**	**X**	**X**	**X**	**X**	**X**	**X**	**X**	**X**	**X**	X					
**10**	**X**	**X**	**X**	**X**	**X**	**X**	**X**	**X**	**X**	**X**	**X**	X	X	X	X	X	X
**11**	X	X	X	X	X												
**12**	X	X	X	X	X												
**13**	X	X	X	X	X												
**14**	X	X	X	X	X												

PCA data analysis
